# Show me the data: A statewide comparative report of National Healthcare Safety Network (NHSN) Antimicrobial Use Option standardized antimicrobial administration ratios (SAARs)

**DOI:** 10.1017/ash.2022.266

**Published:** 2022-07-15

**Authors:** Hana R. Winders, Kayla Antosz, Majdi Al-Hasan, P. Brandon Bookstaver, Pamela Bailey, Katie Waites, Julie Ann Justo

**Affiliations:** 1 Department of Pharmacy, Prisma Health Midlands, Columbia, South Carolina; 2 Department of Clinical Pharmacy & Outcomes Sciences, University of South Carolina College of Pharmacy, Columbia, South Carolina; 3 University of South Carolina School of Medicine, Columbia, South Carolina; 4 Department of Medicine, Prisma Health Midlands, Columbia, South Carolina; 5 South Carolina Department of Health and Environmental Control, Columbia, South Carolina

## Abstract

The Antimicrobial Stewardship Collaborative of South Carolina created quarterly Comparative SAAR Analysis Reports based on standardized antimicrobial administration ratio (SAAR) data from the NHSN Antimicrobial Use (AU) Option. These reports provide SAAR histograms and site-specific feedback to participating facilities in South Carolina. They were created to improve antimicrobial use throughout the state, especially in rural regions.

The Antimicrobial Stewardship Collaborative of South Carolina (ASC-SC) was established in 2016 as a collaboration of antimicrobial stewards focused on optimizing antimicrobial use in academic medical centers, acute-care hospitals, skilled nursing facilities, and ambulatory clinics throughout South Carolina. ASC-SC worked with the South Carolina Department of Health and Environmental Control to obtain access to the National Healthcare Safety Network (NHSN) Antimicrobial Use (AU) Option data for hospitals in South Carolina. The AU Option provides a risk-adjusted estimate of observed-to-expected antibiotic use ratios, otherwise known as the standardized antimicrobial administration ratio (SAAR), a metric developed by the Centers for Disease Control and Prevention (CDC).^1^

## Methods

**Fig. 1.**
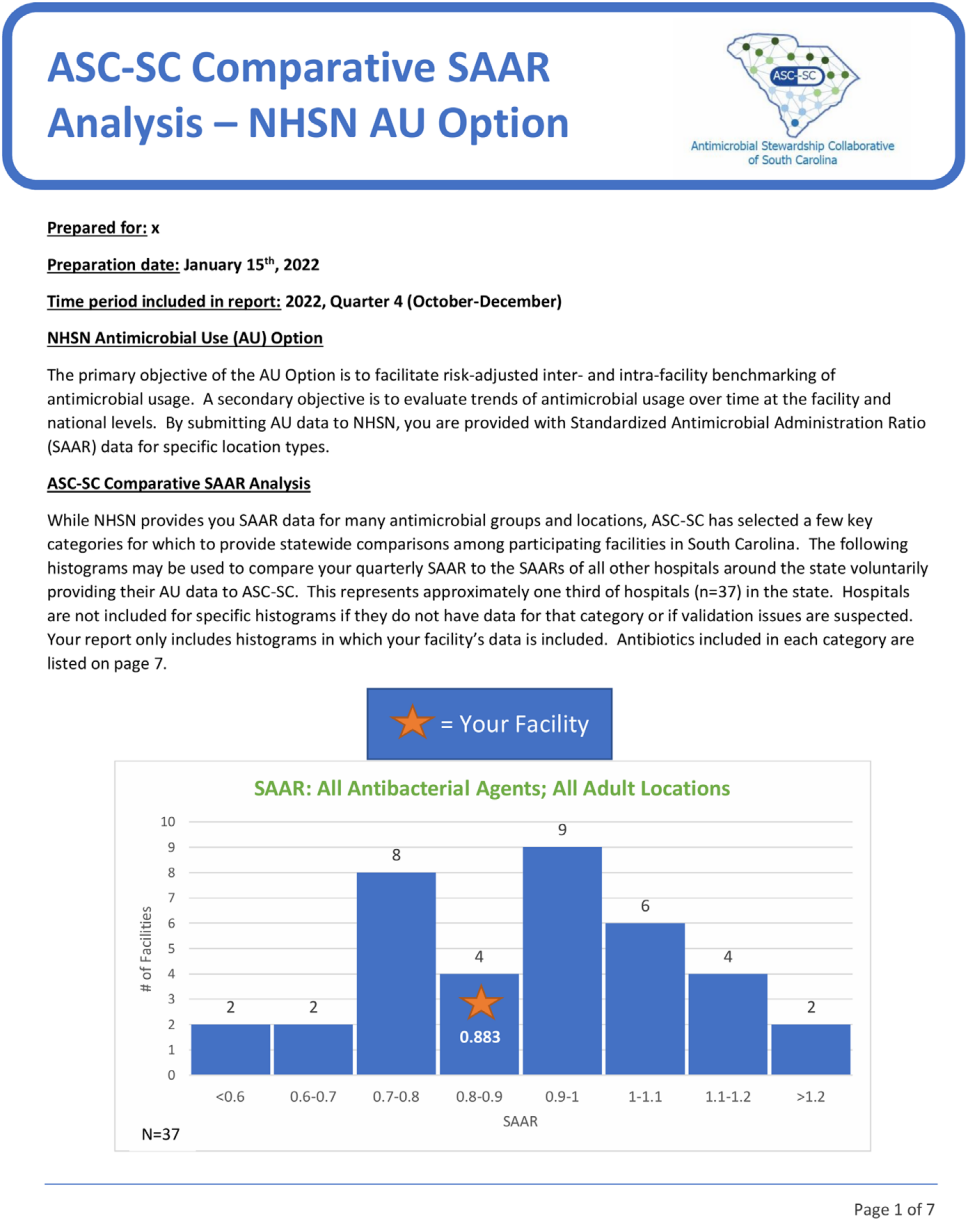
Example of page 1 of the Antimicrobial Stewardship Collaborative of South Carolina (ASC-SC) comparative standardized antimicrobial administration ratio (SAAR) analysis—National Health Safety Network (NHSN) Antimicrobial Use (AU) Option report.

The lead antimicrobial stewardship pharmacist of ASC-SC compiled all statewide SAAR data into a spreadsheet that was used to generate quarterly, individualized, deidentified reports for South Carolina hospitals participating in the AU Option. The reports contained facility name, preparation date, quarter of the year included in the report, a brief overview of the NHSN AU Option and ASC-SC Comparative SAAR Analysis Report, statewide histograms of individual facility SAAR data compared to blinded SAAR data from all facilities, site-specific feedback from ASC-SC for future antimicrobial stewardship initiatives, and information about additional resources (Fig. 1). The following SAARs were used to generate 9 statewide histograms included in the report: all antibacterial agents in all adult SAAR locations, broad-spectrum antibacterial agents predominantly used for hospital-onset infections in adult ICUs and in adult wards, broad-spectrum antibacterial agents predominantly used for community-acquired infections in adult ICUs and in adult wards, narrow-spectrum β-lactam agents in adult ICUs and in adult wards, and antibacterial agents predominantly used for resistant gram-positive infections in adult ICUs and in adult wards. All reports were sent to participating facilities via e-mail.

## Results

The ASC-SC Comparative SAAR Analysis Reports have been generated every quarter since the second quarter of 2019 for each facility in South Carolina that reports antimicrobial use data to the NHSN. In 2021, 144 individualized reports were created. The volume increased from an average of 24 facilities per quarter in 2019 to 36 facilities per quarter in 2021, which represents ∼43% of hospitals in South Carolina that currently report antimicrobial use data to the NHSN. An average of 10 reports per quarter were sent to facilities in rural locations. Additional facility characteristics are listed in Table 1.

**Table 1.** Reporting Facility Characteristics^a^

Characteristic	Facilities (N=37), No. (%)
**Bed size**	
<100 beds	14 (38)
100–400 beds	16 (43)
>400 beds	7 (19)
Rural	10 (27)
Community hospital	34 (92)
Academic medical center	3 (8)

a Based on Quarter 4, 2021 report. Characteristics may change each quarter depending on facilities reporting to NHSN.

The site-specific feedback section offered individual facilities interpretation and guidance from ASC-SC experts regarding individual facility SAAR data reported to NHSN. This section was typically two-thirds of a page to 1 page long, single-spaced. It included detailed comparisons of each SAAR by various domains such as temporal trends within the individual facility (eg, increasing, stable, or decreasing SAAR over time), as well as a commentary on SAAR variability by individual facility location (eg, general medicine ward vs surgical intensive care unit) and/or blinded comparison to other South Carolina facilities with similar bed sizes. In addition, feedback was provided about which antimicrobial stewardship interventions might prove most beneficial for the individual facility. For example, if one ward location had a disproportionally higher SAAR for broad-spectrum antibacterial agents predominantly used for hospital-onset infections and a very low SAAR for narrow-spectrum β-lactam agents, the recommendation would be to re-evaluate efforts to de-escalate patients on these broad-spectrum antibacterial agents on that ward.

During this process, minor NHSN reporting errors were identified and communicated to the respective facilities, improving the accuracy and validity of reported SAAR data over time. Examples of such discrepancies include skipped months of reporting, facility-level relocation of patients, and the presence of antimicrobial days with 0 patient days present. If significant errors were identified, those hospitals were excluded from the corresponding histograms.

## Discussion

The ASC-SC Comparative SAAR Analysis Reports appear to be the first to provide statewide comparative SAAR analyses directly to individual facilities in the United States, and the first to compare SAAR data at the statewide level. Coordination of regional efforts is essential to identifying trends in antimicrobial use and antimicrobial resistance and may be accomplished via various efforts.^2^ Although other states have also published on statewide or local SAAR data, ASC-SC is unique in that it gives these data back to the facility in the context of other SAAR data within South Carolina to assist with stewardship efforts at the institutional level.^3–5^ The SAARs provided directly to each facility by NHSN are inherently comparative in nature, with adjustment for expected antimicrobial use across national facilities of a similar size and type. The CDC published a 2020 Antimicrobial Use Report that facilitates national comparison by SAAR type and location, which is useful for individual facilities to compare their SAAR on a national level.^6^ However, geographical region within the United States is not a factor in the NHSN model. It is possible that regional prevalence of resistant organisms leads to regional differences in antimicrobial use. The ASC-SC Comparative SAAR Analysis Reports facilitates an additional comparison of facility SAAR data across a statewide cohort, which likely shares a more comparable epidemiology of organisms than the national cohort of facilities within the NHSN. Furthermore, histograms were chosen as a visual aid and to provide anonymity among those sharing data with ASC-SC.

Feedback from individual facilities has generally been positive; many have reported that they find the report useful in guiding their antimicrobial stewardship activities. Select facilities stated that they use these reports in quarterly antimicrobial stewardship meetings to create goals in response to SAAR analyses and recommendations provided by ASC-SC. Specifically, facilities appreciate the regional or local comparisons that are more relevant than national data, and they are easily able to compare themselves to hospitals of similar size. These reports are provided at regular intervals, and the graphs they generate can also be used to address the CDC Core Elements of Hospital Antibiotic Stewardship Programs, such as tracking and/or reporting. South Carolina has a high proportion of hospitals (97%) meeting all the CDC 7 Core Elements in both 2019 and 2020.^7^

The collaborative efforts of ASC-SC may also help address healthcare disparities related to lack of access to infectious disease (ID) physicians and pharmacists.^8–10^ In South Carolina, 14 hospitals (15.5%) are in rural areas. Multiple facilities with whom ASC-SC interacts do not have access to ID expertise; thus, they are targeted in ASC-SC’s antimicrobial stewardship outreach.

Potential limitations of these reports include missing data from state facilities not reporting to the NHSN AU Option as well as the lack of comments on other quality measures of interest to antimicrobial stewardship programs (eg, incidence of *Clostridioides difficile* infection, mortality, length of stay, readmission rates). The reports also exclude data on the NHSN Antimicrobial Resistance (AR) Option given the small number of facilities currently reporting these data within the state. Future directions include researching the impact of these reports on antimicrobial stewardship efforts, incorporating antimicrobial resistance data and promoting additional facilities to report to the NHSN AR Option, and incorporating standardized infection ratio (SIR) metrics for healthcare-associated infections into the reports.

In conclusion, the ASC-SC Comparative SAAR Analysis–NHSN AU Option Reports provide benchmarking and expert interpretation of antimicrobial use at an individual facility compared to other facilities within the state of South Carolina. These reports can be utilized to compare antimicrobial use to that of facilities within the same geographical region. Other states may consider adopting a statewide report to improve regional antimicrobial stewardship.
